# Docosahexaenoic acid (DHA) impairs hypoxia-induced cellular and exosomal overexpression of immune-checkpoints and immunomodulatory molecules in different subtypes of breast cancer cells

**DOI:** 10.1186/s40795-024-00844-y

**Published:** 2024-03-04

**Authors:** Sepideh Maralbashi, Cynthia Aslan, Houman Kahroba, Milad Asadi, Mohammad Sadegh Soltani-Zangbar, Navideh Haghnavaz, Farhad Jadidi, Farhad Salari, Tohid Kazemi

**Affiliations:** 1 Applied drug research center, Tabriz University of Medical Sciences, Tabriz, Iran; 2 Department of Immunology, Faculty of Medicine, Kermanshah University of Medical Science, Kermanshah, Iran; 3 Immunology Research Center, Tabriz University of Medical Science, Tabriz, Iran; 4 Student Research Committee, Tabriz University of Medical Science, Tabriz, Iran; 5 Department of Toxicogenomics, GROW School for Oncology and Developmental Biology, Maastricht University, Maastricht, the Netherlands; 6 Centre for Environmental Sciences, Hasselt University, Hasselt, Belgium; 7 Department of Basic Oncology, Health Institute of Ege University, Izmir, Turkey; 8 Department of Immunology, Faculty of Medicine, Tabriz University of Medical Science, Tabriz, Iran

**Keywords:** Docosahexaenoic acid, Breast cancer, Exosome, Immune-checkpoints, MicroRNA

## Abstract

**Background:**

Tumor cells express immune-checkpoint molecules to suppress anti-tumor immune responses. In part, immune evasion takes place by secreting exosomes bearing immune-checkpoint and immunomodulatory molecules and their inducing and/or regulating agents e.g., microRNAs (miRs). This study aimed to evaluate the effects of omega-3 fatty acid, docosahexaenoic acid (DHA), on the expression of some selected immune-checkpoint and immunomodulatory molecules and their regulating miRs under both normoxic and hypoxic conditions in triple negative (TNBC) invasive and triple positive non-invasive breast cancer cell lines.

**Methods:**

MDA-MB-231 and BT-474 cells were treated with 100 µM DHA under hypoxic and normoxic conditions for 24 h. Exosomes were isolated by ultracentrifuge and confirmed by electron microscope and anti-CD9, -CD63, -CD81 immunoblotting. Total RNA from cells and exosomes were extracted and expression of CD39, CD73, CD47, CD80, PD-L1, B7-H3, B7-H4 genes and their related miRs were evaluated by quantitative Real-time PCR.

**Results:**

This study showed significant over-expression of immune-checkpoint and immunomodulatory molecules under hypoxic condition. Treatment with DHA resulted in a significant decrease in immune-checkpoint and immunomodulatory molecule expression as well as an upregulation of their regulatory miRNA expression.

**Conclusion:**

DHA supplementation may be utilized in breast cancer therapy for down-regulation of cellular and exosomal immune escape-related molecules.

**Supplementary Information:**

The online version contains supplementary material available at 10.1186/s40795-024-00844-y.

## Significance of the study

This study showed anti-immunosuppressive effect of DHA on BC cell lines in normoxic and hypoxic conditions.

## Introduction

Globally, breast cancer (BC) is considered the most prevalent type of malignancy as well as the second leading cause of cancer-related deaths [1]. BCs are classified according to their expression patterns of certain molecules including estrogen receptor (ER), progesterone receptor (PR), and human epidermal growth factor receptor 2 (HER2) [2]. Triple-positive BC (TPBC) subtype, also known as luminal B, accounts for almost 40% of cancer-related deaths [3] and triple-negative BC (TNBC) is responsible for 10–20% of all diagnosed BCs [4, 5]. The TNBC subtype is known as the most aggressive subtype with high recurrence rates, resulting in a higher death rate during the first year after treatment [6, 7].

Several studies have demonstrated the anti-cancer effects of omega-3 long chain polyunsaturated fatty acids (n–3 LCPUFAs), particularly DHA, on BC in vivo and in vitro [8, 9]. However, only a few studies have investigated DHA’s application for treating BC by reducing the tumor’s immune evasion [10, 11]. Accurate equilibrium between immune activation and suppression is called immune homeostasis. It not only accomplish efficient recognition, and eradication of pathogen but also inhibit self-destruction immune reactions. The evidences demonstrate that defeat of immune homeostasis occures in most of cancers [12]. When inflammatory responses are produced by immune system to fight infections, immune checkpoints play vital role to secure tissues from harm. Cancer cells highly express immune checkpoint molecules result in tumors escape from the antitumor immune response. These molecules are ligand-receptor pairs that have stimulatory or inhibitory effect on immune responses. Immune checkpoints are expressed on cancer cells, antigen-presenting cells, immune cells, or other sorts of cells cause development of immune system [12]. Cytotoxic T lymphocyte associated protein 4 (CTLA4) is an immune-checkpoint molecule on T cells which interactes with CD80 (B7-1) and CD86 (B7-2) on tumor cells to inhibit the induction phase of the T cell response. Of them, interaction of programmed cell death protein 1 (PD-1) and its ligand, PD-L1, inhibits effector phase of the T cell response [13]. Additional immune-checkpoints that may also contribute to tumor immune escape have been introduced such as CD47, B7-H3 (CD276), B7-H4 (VTCN1,B7X), CD39 and CD73 [14, 15]. CD39 and CD73 are two ectonucleosidases that are expressed on tumor cell surface, and have important roles in createing an immunosuppressive tumor microenvironment [12]. These ectonucleosidases convert ATP to adenosine which can inhibit the T lymphocyte effector activity through the adenosine receptor [16]. CD47 is another immune-regulatory target present on the surface of various cells including solid and hematologic tumors. It signals circulating immune cells e.g. macrophages “not to eat” them by binding to phagocyte expressed signal regulatory protein-alpha (SIRPα) [17]. B7-H3 and B7-H4 are known as immunomodulatory proteins which belong to ligands of the B7 family which indicate inhibitory activity by suppressing T cells proliferation and activation and also downregulation of the T cells response. Therefore these molecules can effectively modulate tumor immunogenicity and cancer progression [18].

Lately, studies have suggested exosomes as novel mediators in immune evasion of tumor cells [19–21]. They are nanovesicles 30–150 nm in diameter and are able to modify immune responses [22, 23]. Exosomes play important roles in cancer development, progresssion and invasiveness by harboring various cargoes specially microRNAs [24, 25]. Apparently, exosomes transfer microRNAs successfully into “target” cells, which may promote the reprogramming of the immune system in favor of cancer development. Shuttled microRNAs serve as important messengers for transferring information between tumor cells, the immune system, and the microenvironment [21].

The purpose of this study was to investigate the effect of DHA on the expression of CD39, CD73, CD47, CD80, PD-L1, B7-H3, B7-H4 genes and miR-142-3p, miR-133a, miR-30a-5p, miR-194, miR-424, miR-155-5p, miR-143 both in MDA-MB-231 (as a metastatic TNBC cell line) and BT-474 (as a non-metastatic triple positive cell line) and also their secreted exosomes under hypoxic and normoxic conditions.

## Materials and methods

### Cell culture and treatments

Two human breast cancer cell lines (BT-474 and MDA-MB-231) were used in this study which were purchased from the National Cell Bank of Iran (Pasteur-Institute, Tehran, Iran). These cell lines were cultured in complete Roswell Park Memorial Institute (RPMI) 1640 medium (Gibco Inc., El Paso, TX) which was supplemented by 10% of fetal bovine serum (Gibco Inc.), also 100 unit/ml penicillin, and 100 µg/ml streptomycin. Cells were incubated at 37 °C in a 95% humidified incubator with 5% CO_2_. Cells with 80% confluency were treated with 100 µM DHA (Sigma-Aldrich) and 25 µM CoCl_2_ for 24 h. Cells treated with vehicle buffer (bovine serum albumin) were considered as control cells.

### Exosome isolation

Once the cells reached 80% confluency, the supernatant was completely removed and the remained cells were washed by RPMI 1640 medium and the cell culture was proceeded by RPMI 1640 medium which was supplemented with the 5% of exosome-free-FBS for 24 h. Subsequently, the supernatants were subjected to centrifugation at 500 g for 30 min (4˚C) (Hettich Rotanta 46R, German) to eliminate cells and debris. Isolation process was followed by 30,000 g centrifugation for 2 h (4˚C) to eliminate the microvesicles and filtration by membrane filter (0.22 μm) and then ultracentrifugation at 110,000 g (4˚C) for 2 h (TLA-100.3, Beckman Coulter Life Sciences, Indiana, US). The exosome pellet was sonicated in PBS 3 times for 30 s and then stored at -20 °C for further experiments.

### Transmitting electron microscope (TEM)

Evaluation of exosome morphology was assessed by transmission electron microscope (TEM). A droplet of exosome containing solution was used to fix the exosomes on 300 mesh copper grid at ambient temperature for 5 min. Then, negative staining was performed with 2% (wt/v) uranyl acetate solution (TAAB, England) for 5 min and visualized by LEO 906 Zeiss instrument with 80 Kv accelerating voltage [26].

### Western blot analysis

Exosomes lysate were prepared by using protein lysis buffer (RIPA, Sigma-Aldrich). Prior to loading on 12% sodium dodecyl sulphate polyacrylamide gel electrophoresis (SDS-PAGE), the exosomal proteins were incubated with β-mercaptoethanol containing loading buffer for 10 min at 70˚C. Exosomal markers were detected by specific antibodies including anti-CD9, -CD81, and -CD63 antibodies (SANTA CRUZ,California, USA) according to standard western blotting protocol [9].

### Quantitative real-time PCR

Cell lines and isolated exosomes were used for RNA extraction by TRIzol method (RiboEX, GeneAll Biotechnology, Korea) according to the instruction of manufacturer. The RNA concentration was evaluated by NanoDrop Spectrophotometer (NanoDrop Technologies, Wilmington, DE, USA) and the RNA stored at -80 °C until next use. The extracted RNA samples were applied to assess the extraction qualitatively by electrophoresis on agarose gel to observe 18 and 28 s ribosomal RNA bands. The miRNAs were reverse transcribed by by Universal cDNA Synthesis Kit II (Exiqon, Vedbaek, Denmark) based on the manufacturer’s instruction. Also, cDNA from RNAs was synthesized by BioFact Kit (Daejeon, Korea).

Evaluation of the expression levels of CD47, CD73, CD39, PD-L1, CD80, B7-H3 and B7-H4 mRNAs, and miR-133a, miR-194, miR-30a-5p, miR-142-3p, miR-155-5p, miR-424, miR-143 was performed by Quantitative Real-time PCR method by using SYBR Green master mix (Ampliqon, Odense, Denmark) on LightCycler 96 system (Roche Company, Basel, Switzerland). Primers for target genes and GAPDH, as the reference gene, were designed by Primer Express 3.0 (Applied Biosystems, Foster City, CA, USA), (Table 1). The comparative C_T_ method was employed to evaluate the relative amounts (using 2^−∆∆CT^formula) of target mRNA in the test samples, which were normalized against GAPDH transcript level. Also, the expression levels of miRNAs were normalized against U6 as the housekeeping gene.

**Table 1:** Primer sequences for studied genes

Target	Primer sequences
CD39	F5’-AGTGATTCCAAGGTCCCAGCA-3’ R5’-CTGGCACCCTGGAAGTCAAAG-3’
CD47	F5^’^-GCCTTGTCCCTATTGTGGCTT-3’ R5’-CTTGGTTTGTGACTGCCCCAT-3’
CD73	F5’-CCAGTCCACTGGAGAGTTCCT-3’ R5’-ACTCGACACTTGGTGCAAAGA-3’
CD80	F5’-AAGAAGTGGCAACGCTGTCCT-3’ R5’-CGGTTCTTGTACTCGGGCCAT-3’
PD-L1	F5’-CTGCAGGGCATTCCAGAAAGA-3’ R5’- TGCGTTCAGCAAATGCCAGTA-3’
B7-H3 (CD276)	F5’-TGACCACATCACCACCCTCTT-3’ R5’-AGCAGGGCTGAACATGATCAC-3’
B7-H4	F5’-ATTTCAGCCTTCAGCATGCCG-3’ R5’-GGTCAACTTGGGATGCCCAGA-3’
GAPDH	F5’- CAAGATCATCAGCAATGCCTCC-3’ R 5’-GCCATCACGCCACAGTTTCC − 3’

### Statistical analysis

The obtained Data was analyzed by using the GraphPad Prism software version 6.0.1 (Graph Pad Prism, San Diego, CA, USA). After calculation of normality of data distribution using the one-way ANOVA test, the group comparisons were investigated using Dunnett and Tukey nonparametric test. The data were represented as mean ± standard deviation (SD) and *P* < 0.05 was considered the level of significance.

## Results

### Exosome characterization

**Fig. 1:**
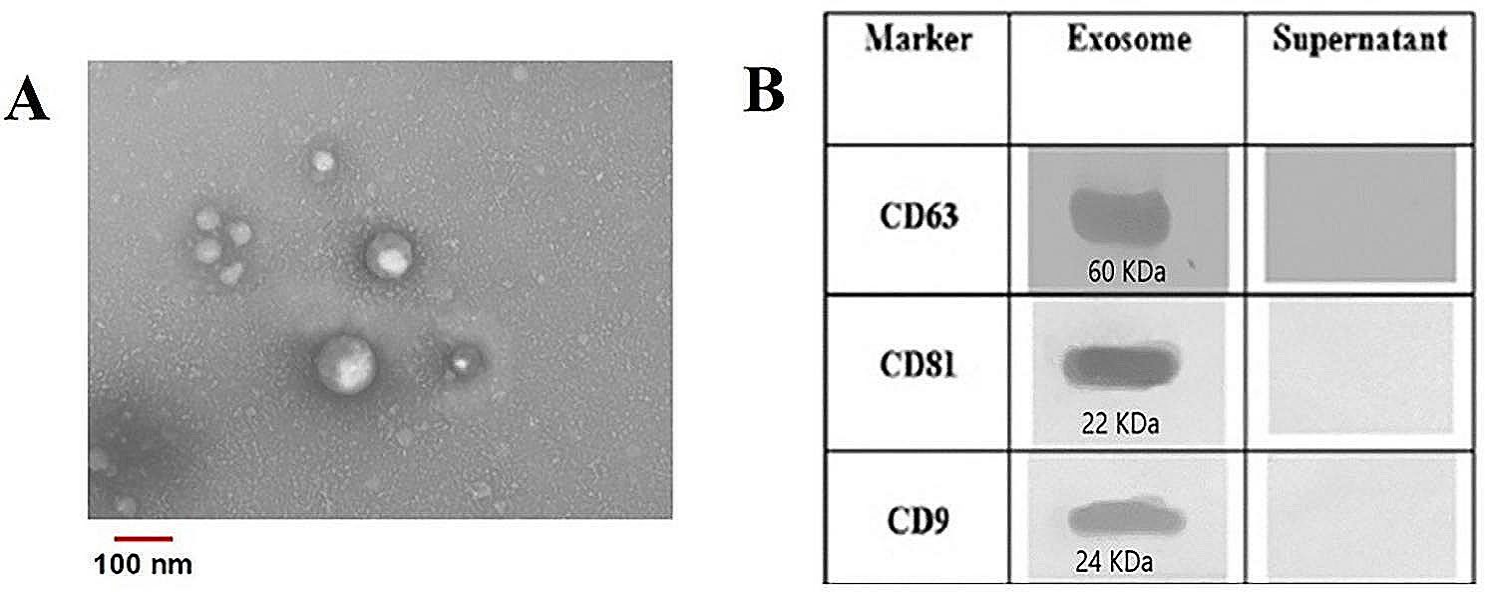
(**A**) TEM result confirms cup-shape for morphology of exosomes around 100 nm, and (**B**) specific protein markers for exosome and supernatant CD9, CD81, and CD63

Isolated Exosomes characteristics have been displayed in Fig. 1 for morphology and specific protein markers. Transmission electron microscope (TEM) results confirmed the morphology and size of the exosomes, and western blotting analysis showed their specific markers including CD9, CD81, and CD63 (Fig. 1.A, B).

### Altered expression levels of target genes in BC cell lines under DHA treatment, and hypoxic condition

**Fig. 2:**
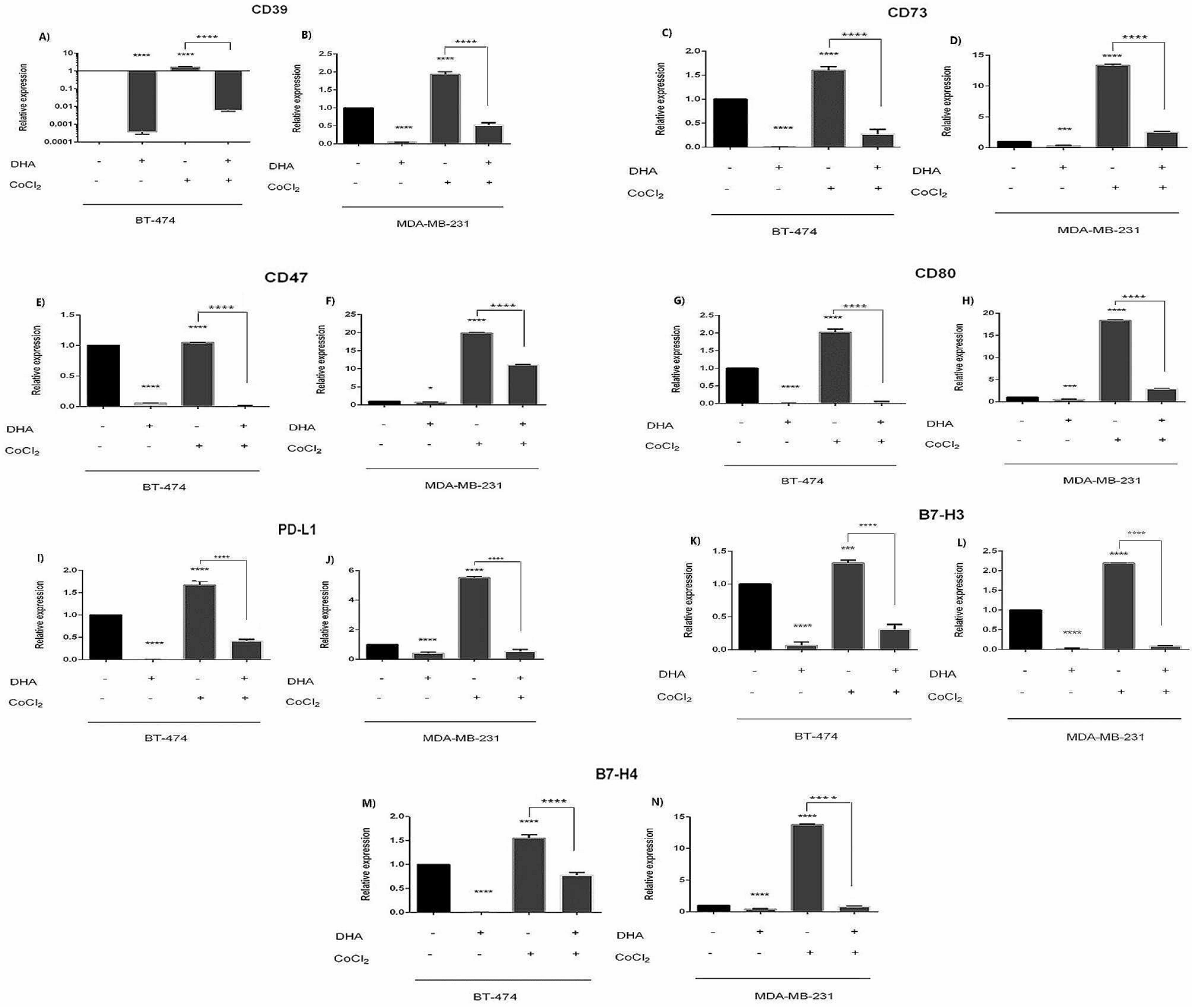
Expression levels of target genes in BC cell lines after treatment with DHA in MDA-MB-231 and BT-474. Cell lines were treated with 100µM under both hypoxic and normoxic conditions for 24 h. Expression level of selected mRNAs were determined by quantitative Real-time PCR * *p* = 0.0324, *** *p* < 0.0006, ****p < 0.0001

Under normoxic condition, treatment of MDA-MB-231 and BT-474 with 100 µM DHA caused significant down-regulation in the expression of CD39, CD73, CD47, CD80, PD-L1, B7-H3, B7-H4 genes. Generally, the influence of DHA on the expression of studied genes were more prominent in BT-474 compared to MDA-MB-231 with the most altered expression level for CD39 in BT-474 (2674fold) and the least down-regulation for CD47 in MDA-MB-231 (1.24fold) (Fig. 2; Tables 2 and 3).

Also, induction of hypoxic condition using 25 µM CoCl_2_ resulted in significant up-regulation in the expression of CD39, CD73, CD47, CD80, PD-L1, B7-H3, B7-H4 genes especially in MDA-MB-231. Interestingly, the most up-regulated gene was CD47 in MDA-MB-231 (20 fold) and the least altered expression level was also for CD47 in BT-474 (1.04 fold) (Fig. 2; Tables 2 and 3).

To explore the effect of DHA in hypoxic condition, cells were treated with both DHA and CoCl_2_.

Statistical analysis showed that all cells down-regulated the expression level of studied genes after treatment with DHA; more altered expression level was observed for CD39, CD73, CD47, and CD80 in BT-474 cells and PD-L1, B7-H3, B7-H4 in MDA-MB-231 (Fig. 2; Tables 2 and 3).

**Table 2:** Expression of studied genes in BT-474 cell line

BT-474						
Normoxic			Hypoxic			
Treated with DHA			Treated with CoCl_2_		Treated with DHA and CoCl_2_	
	Fold change	*P*-value *	Fold change	*P*-value **	Fold change	*P*-value***
CD39	-2674	< 0.0001	1.657001	< 0.0001	-277	< 0.0001
CD73	-567.85	< 0.0001	1.599488	< 0.0001	-5.85	< 0.0001
CD47	-18.00018	< 0.0001	1.046463	< 0.0001	-96.11	< 0.0001
CD80	-211.014	< 0.0001	2.024291	< 0.0001	-57.74	< 0.0001
PD-L1	-347.101	< 0.0001	1.674481	< 0.0001	-4	< 0.0001
B7-H3	-15.156	< 0.0001	1.320132	0.0001	-4.25	< 0.0001
B7-H4	-490.196	< 0.0001	1.548227	< 0.0001	-1.99	< 0.0001

*: compared control with DHA treated group

**: compared control with CoCl_2_ treated group

***: compared CoCl_2_ treated group with DHA + CoCl_2_ treated group

**Table 3:** Expression of studied genes in MDA-MB-231 cell line

MDA-MB-231						
Normoxic			Hypoxic			
Treated with DHA			Treated with CoCl_2_		Treated with DHA and CoCl_2_	
	Fold change	*P*-value*	Fold change	*P*-value**	Fold change	*P*-value***
CD39	-21.54	< 0.0001	1.931994	< 0.0001	-3.70	< 0.0001
CD73	-3.2329	0.0001	13.36898	< 0.0001	-5.25	< 0.0001
CD47	-1.2378	0.0324	19.86097	< 0.0001	-1.78	< 0.0001
CD80	-2.1281	0.0006	18.38235	< 0.0001	-6.32	< 0.0001
PD-L1	-2.4426	< 0.0001	5.52975	< 0.0001	-10.27	< 0.0001
B7-H3	-40.98	< 0.0001	2.191541	< 0.0001	-24.91	< 0.0001
B7-H4	-2.4056	< 0.0001	13.7931	< 0.0001	-16.91	< 0.0001

*: compared control with DHA treated group

**: compared control with CoCl_2_ treated group

***: compared CoCl_2_ treated group with DHA + CoCl_2_ treated group

### Altered expression levels of target miRNAs in BC cell lines under hypoxic condition, and DHA treatment

**Fig. 3:**
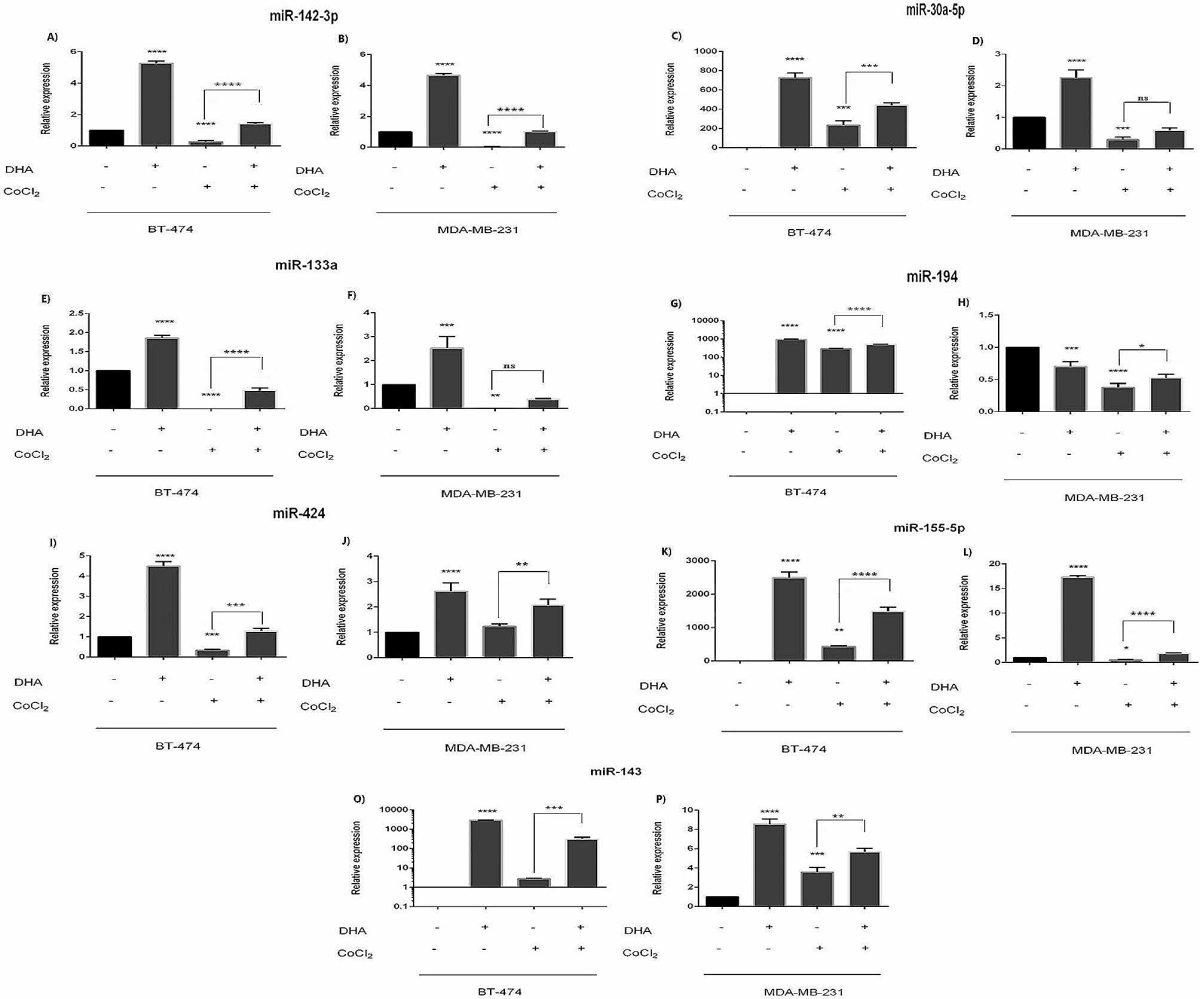
Expression levels of studied miRs in BC cell lines after treatment with DHA in MDA-MB-231 and BT-474. Cell lines were treated with 100µM under both hypoxic and normoxic conditions for 24 h. Expression level of selected mRNAs were determined by quantitative Real-time PCR * *p* = 0.0465, ** *p* < 0.0030, *** *p* < 0.0042, **** *p* < 0.0001

Under normoxic conditions, up-regulation of tumor-suppressor miRs was observed in MDA-MB-231 and BT-474 treated with DHA, including miR-142-3p, miR-30a-5p, miR-133a, miR-194, miR-424, miR-155-5p, miR-143 (Fig. 3; Tables 3 and 4). These miRs control the expression of CD39, CD73, CD47, PD-L1, CD80, PD-L1, and B7-H3, B7-H4 respectively. Likewise, DHA caused tumor-suppressor miRs to be up-regulated under hypoxic conditions compared with CoCl2 alone in MDA-MB-231 and BT-474 (Fig. 3; Tables 4 and 5).

In both MDA-MB-231 and BT-474 cell lines, treatment with 25 µM CoCl2 induced hypoxia which resulted in non-consistent and different patterns of altered miR expression levels. (Fig. 3; Tables 4 and 5).

**Table 4:** Expression level of miRNAs in BT-474 cell line

BT-474						
Normoxic			Hypoxic			
Treated with DHA			Treated with CoCl_2_		Treated with DHA and CoCl_2_	
	Fold change	*P*-value*	Fold change	*P*-value**	Fold change	*P*-value***
MiR-142	5.268704	< 0.0001	-3.6217	< 0.0001	5.263158	< 0.0001
MiR-30a	769.2308	< 0.0001	238.0952	0.0001	1.923077	0.0004
MiR-133a	1.85701	< 0.0001	-7092.19	< 0.0001	3448.276	< 0.0001
MiR-194	952.381	< 0.0001	292.8258	< 0.0001	1.785714	< 0.0001
MiR-424	4.496403	< 0.0001	-3.0065	0.0007	4	0.0001
MiR-155	2500	< 0.0001	432.9004	0.0026	3.571429	< 0.0001
MiR-143	2941.176	< 0.0001	2.765487	> 0.9999	113.6364	0.0004

*: compared control with DHA treated group

**: compared control with CoCl_2_ treated group

***: compared CoCl_2_ treated group with DHA + CoCl_2_ treated group

**Table 5:** Expression level of miRNAs in MDA-MB-231 cell line

MDA-MB-231						
Normoxic			Hypoxic			
Treated with DHA			Treated with CoCl_2_		Treated with DHA and CoCl_2_	
	Fold change	*P*-value*	Fold change	*P*-value**	Fold change	*P*-value***
MiR-142	4.657662	< 0.0001	-24.720	< 0.0001	25.64103	< 0.0001
MiR-30a	2.254283	< 0.0001	-3.3951	0.0006	2.040816	0.0999
MiR-133a	2.524615	0.0002	-128.2544	0.0030	50	0.3133
MiR-194	-1.4239	0.0005	-2.666	< 0.0001	1.408451	0.0465
MiR-424	2.615063	< 0.0001	1.230769	0.4314 NS	1.694915	0.0047
MiR-155	17.33102	< 0.0001	-1.9293	0.0153	3.703704	< 0.0001
MiR-143	8.547009	< 0.0001	3.575259	0.0002	1.612903	0.0011

*: compared control with DHA treated group

**: compared control with CoCl_2_ treated group

***: compared CoCl_2_ treated group with DHA + CoCl_2_ treated group

### Altered expression levels of target genes in exosomes-derived from BC cell lines under hypoxic condition, and DHA treatment

**Fig. 4:**
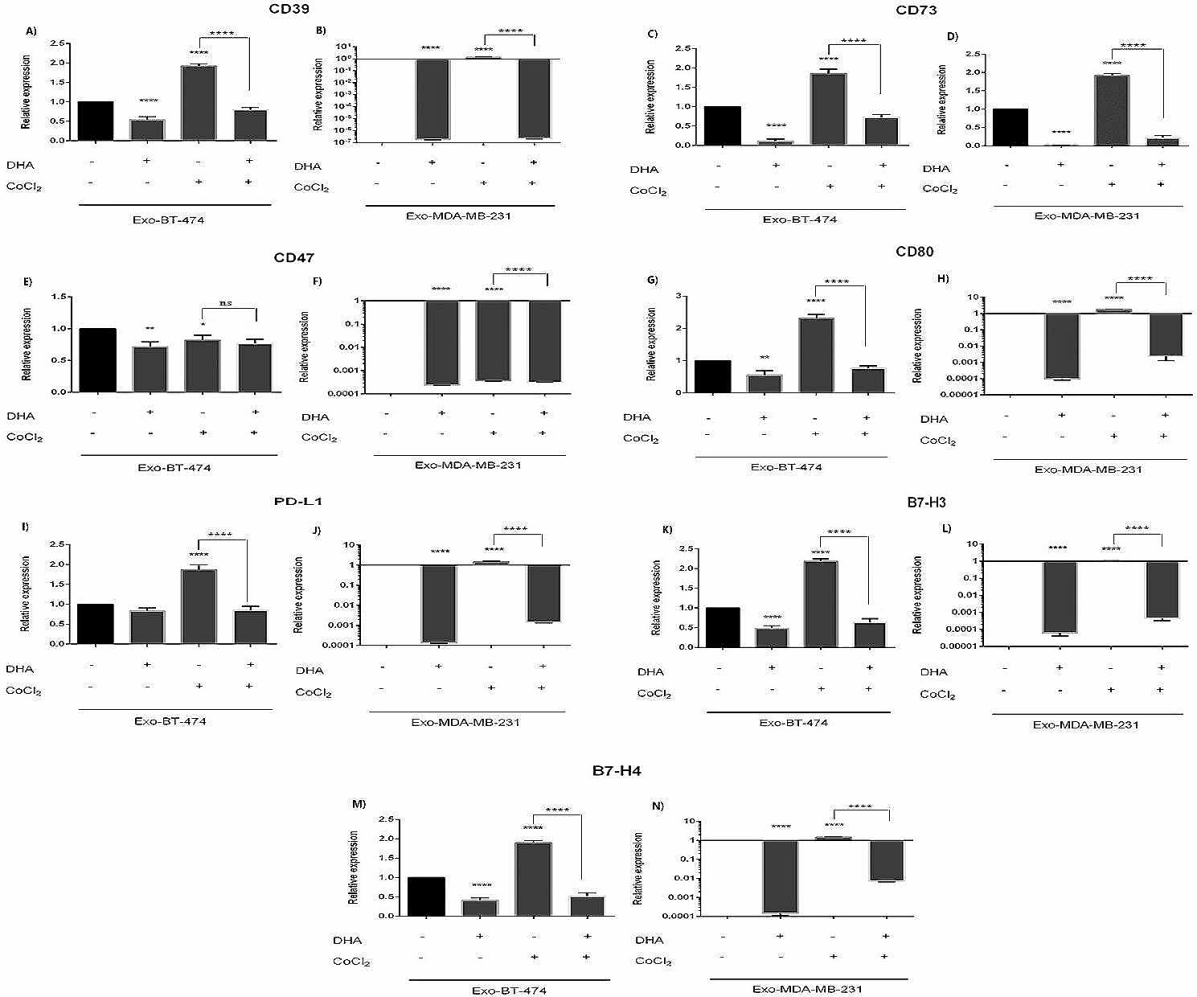
Expression levels of target genes in exosomes-derived from BC cell lines after treatment with DHA in MDA-MB-231 and BT-474. Cell lines were treated with 100µM under both hypoxic and normoxic conditions for 24 h. Expression level of selected mRNAs were determined by quantitative Real-time PCR * *p* = 0.0240, ** *p* < 0.0160, **** *p* < 0.0001

A significant reduction of gene expression (CD39, CD73, CD47, CD80, B7-H3, and B7-H4) was observed in exosomes derived from BT-474 and MDA-MB-231 cells treated with DHA under normoxic conditions. This effect was notable in exosomes derived from MDA-MB-231 (Fig. 4; Tables 6 and 7). On the other hand, hypoxic condition induced by CoCl_2_ led to the significant over-expression of all studied genes in exosomes purified from BT-474 and MDA-MB-231. Interestingly, only CD47 gene was under-expressed in exosomes derived from both cell lines under hypoxic condition. Again, this effect was prominent in MDA-MB-231 cell lines (Fig. 4; Tables 6 and 7).

DHA in hypoxic condition downregulated the expression of all studied genes in exosomes derived from BT-474 and MDA-MB-231 cell lines, consistent with other results. (Fig. 4; Tables 6 and 7).

**Table 6:** Expression of studied genes in exosomes- derived BT-474 cell line

Exo-BT-474						
Normoxic			Hypoxic			
Treated with DHA			Treated with CoCl_2_		Treated with DHA and CoCl_2_	
	Fold change	*P*-value*	Fold change	*P*-value**	Fold change	*P*-value***
CD39	-1.8856	< 0.0001	1.924928	< 0.0001	-2.41	< 0.0001
CD73	-8.98	< 0.0001	1.85701	< 0.0001	-2.54	< 0.0001
CD47	-1.39	0.0016	-1.21	0.0240	-1.07	0.7316
CD80	-1.79	0.0012	2.309469	< 0.0001	-3.02	< 0.0001
PD-L1	-1.19	0.1186	1.869858	< 0.0001	-2.20	< 0.0001
B7-H3	-2.064	< 0.0001	2.184837	< 0.0001	-3.44	< 0.0001
B7-H4	-2.428	< 0.0001	1.897533	< 0.0001	-3.66	< 0.0001

*: compared control with DHA treated group

**: compared control with CoCl_2_ treated group

***: compared CoCl_2_ treated group with DHA + CoCl_2_ treated group

**Table 7:** Expression of studied genes in exosomes- derived MDA-MB-231 cell line

Exo-MDA-MB-231						
Normoxic			Hypoxic			
Treated with DHA			Treated with CoCl_2_		Treated with DHA and CoCl_2_	
	Fold change	*P*-value*	Fold change	*P*-value**	Fold change	*P*-value***
CD39	-5,376,344	< 0.0001	1.364815	< 0.0001	-6,170,426	< 0.0001
CD73	-116.6	< 0.0001	1.920861	< 0.0001	-8.86	< 0.0001
CD47	-4149.3	< 0.0001	-2695.4	< 0.0001	-1.18	< 0.0001
CD80	-10,416	< 0.0001	1.739433	< 0.0001	-5516	< 0.0001
PD-L1	-7246.3	< 0.0001	1.488982	< 0.0001	-1021	< 0.0001
B7-H3	-16181.2	< 0.0001	1.044386	< 0.0001	-2373	< 0.0001
B7-H4	-6711.4	< 0.0001	1.465201	< 0.0001	-199	< 0.0001

*: compared control with DHA treated group

**: compared control with CoCl_2_ treated group

***: compared CoCl_2_ treated group with DHA + CoCl_2_ treated group

### Altered expression levels of target miRNAs in exosomes derived from BC cell lines under hypoxic condition, and DHA treatment

**Fig. 5:**
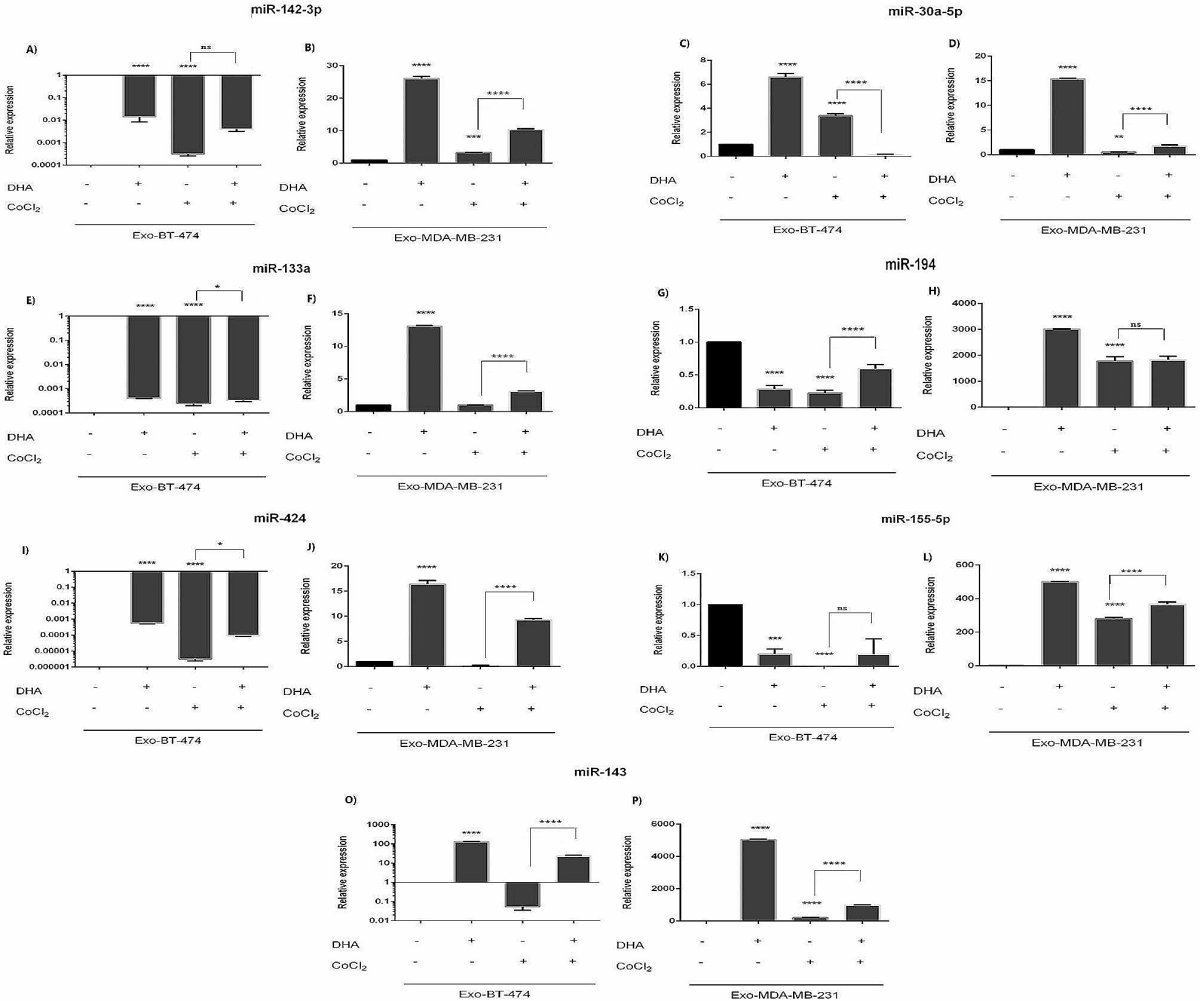
Expression levels of studied miRs in exosomes-derived from BC cell lines after treatment with DHA in MDA-MB-231 and BT-474. Cell lines were treated with 100µM under both hypoxic and normoxic conditions for 24 h. Expression level of selected mRNAs were determined by quantitative Real-time PCR * *p* < 0.0392, ** *p* < 0.0071, *** *p* = 0.0002, **** *p* < 0.0001

Expression pattern of selected miRNAs in exosomes derived from MDA-MB-231 and BT-474 cell lines after DHA treatment showed that all miRs were up-regulated in MDA-MB-231 in both hypoxic and normoxic conditions (exception was miR-194 in combination of DHA and CoCl_2_ treatment); however, only miR-30a and miR-143 showed higher expression in BT-474 after DHA treatment in normoxic condition. (Fig. 5; Tables 8 and 9). The expression of all tumor suppressor miRs in BT-474-derived exosomes was down-regulated under hypoxic condition (Fig. 5; Tables 8 and 9).

**Table 8:** Expression level of miRNAs in exosomes-derived BT-474 cell line

Exo-BT-474						
Normoxic			Hypoxic			
Treated with DHA			Treated with CoCl_2_		Treated with DHA and CoCl_2_	
	Fold change	*P*-value*	Fold change	*P*-value**	Fold change	*P*-value***
MiR-142	-71.46	< 0.0001	-3154.57	< 0.0001	12.34568	0.4330
MiR-30a	6.60502	< 0.0001	3.386387	< 0.0001	-27.64	< 0.0001
MiR-133a	-2444.98	< 0.0001	-4032.25	< 0.0001	1.315789	0.0392
MiR-194	-3.5385	< 0.0001	-4.4471	< 0.0001	2.631579	< 0.0001
MiR-424	-1785.71	< 0.0001	-315457.4	< 0.0001	29.41176	0.0115
MiR-155	-4.996	0.0002	-442.67	< 0.0001	90.90909	0.3112
MiR-143	131.2336	< 0.0001	-18.3297	0.8760	434.7826	< 0.0001

*: compared control with DHA treated group

**: compared control with CoCl_2_ treated group

***: compared CoCl_2_ treated group with DHA + CoCl_2_ treated group

**Table 9:** Expression level of miRNAs in exosomes-derived MDA-MB-231 cell line

Exo-MDA-MB-231						
Normoxic			Hypoxic			
Treated with DHA			Treated with CoCl_2_		Treated with DHA and CoCl_2_	
	Fold change	*P*-value*	Fold change	*P*-value**	Fold change	*P*-value***
MiR-142	26.02811	< 0.0001	3.238342	0.0002	3.225806	< 0.0001
MiR-30a	15.38462	< 0.0001	-1.8946	0.0071	3.333333	< 0.0001
MiR-133a	13.07702	< 0.0001	1.01688	0.9806	3.030303	< 0.0001
MiR-194	3030.303	< 0.0001	1785.714	< 0.0001	1.030928	0.9508
MiR-424	16.42036	< 0.0001	-6.9105	0.0659	62.5	< 0.0001
MiR-155	502.5126	< 0.0001	281.6901	< 0.0001	1.298701	< 0.0001
MiR-143	5263.158	< 0.0001	219.7802	< 0.0001	4.545455	< 0.0001

*: compared control with DHA treated group

**: compared control with CoCl_2_ treated group

***: compared CoCl_2_ treated group with DHA + CoCl_2_ treated group

## Discussion

Newly, emerging strategy for immunotherapy of cancer is manipulation of inhibitory mechanisms of cancer cells on anti-tumor immune responses. Such mechanisms are exerted via some membrane bound and secretory molecules produced by tumor cells, that upon binding to their own receptors can initiate inhibitory signaling in immune cells. Exosomes are also recently discovered additional pathway for delivery of such inhibitory cargo including proteins, mRNAs and miRs. Hannafon et al. indicated that DHA changed BC exosome secretion and microRNA contents, which inhibited angiogenesis [27]. Aslan et al. proved that DHA has anti-angiogenesis effect in cellular and exosomal levels in BC cell lines [28]. Suppressing metastasis by DHA in TNBC cell line in normoxic and hypoxic conditions have been shown by Javadian et al. [9]. In another study, pro-apoptotic effect of DHA has been demonstrated via increasing ratio of cyclic AMP/cyclic GMP levels, and elevated the expression of peroxisome proliferator activated receptor (PPAR)-α and toll-like receptor 4 (TLR-4) in treated BC tissues [29]. Huang et al. indicated that DHA inhibited proliferation of MCF-7 BC cell line by suppressing pAkt signaling [30]. About immune-activating effect of DHA, Han et al., indicated that DHA could activate and increase the proliferation and phagocytosis activity of macrophages, increase the proliferation of spleen cells and the natural killer (NK) cells activity, improve the production of cytokines including interleukin (IL)-1β, IL-2, tumor necrosis factor (TNF)-α and interferon (IFN)-γ in the spleen of immunosuppressed BALB/c mice [31].

The aim of this study was to investigate possible beneficial effect of omega-3 fatty acid DHA on the expression levels of immune checkpoint and immunomodulatory molecules and their controlling miRs in two metastatic and non-metastatic breast cancer cell lines and their secreted exosomes under both hypoxic and normoxic conditions. Our results showed that hypoxia led to the up-regulated cellular and exosomal expression of CD39, CD73, CD47, CD80, PD-L1, B7-H3, and B7-H4 genes in BT-474 and MDA-MB-231 cell lines (Figs. 2 and 4; Tables 2, 3, 6 and 7). Treatment of cells with DHA down-regulated cellular and exosomal expression of all studied genes undernormoxic condition (Figs. 2 and 4; Tables 2, 3, 6 and 7).

In this study, we noticed that DHA down-regulated the expression of CD39 and CD73 in two BC cells and their derived exosomes under hypoxic and normoxic conditions. Thom et al. reported that CD39 activity was weaken after treatment with DHA in endothelial cells [32]. Their observations about expression of CD73 was increased in protein level but in mRNA level didn’t change after treatment with DHA [32]. Clayton et al. reported the first study of the immunosuppressive role of exosomes by expressing CD39 (also known as ectonucleoside triphosphatediphosphohydrolase-1) and CD73 (also known as ecto-5ʹ-nucleotidase) [33]. CD39 and CD73 are ecto-enzymes that promote tumor immune escape by the production of immunosuppressive extracellular adenosine in the tumor microenvironment and intervene with the anti-tumor immune reactions [34, 35]. Theodoraki et al. demonstrated that the highest levels of CD39/CD73 ectoenzymes and adenosine production were found in tumor-derived exosomes in patients with the stages III/IV head and neck squamous cell carcinoma [36]. Increased levels of CD73 expression on epithelial tumor cells were quietly associated with decreased disease-free survival (DFS), overall survival (OS) and negatively correlated with the anti-tumor immunity in human TNBC [34]. The decreased expressions of CD39 and CD73 after treatment of DHA are harmful for tumor growth.

We observed DHA decreased CD47 expression in both cell lines whereas in exosomes alteration of CD47 expression between normoxic and hypoxic conditions didn’t show strike differences. Zhang et al. proved that hypoxia increased hypoxia-inducible factor 1 (HIF-1)-mediated expression of CD47 in BC cells and led to reduction of phagocytosis BC cancer cells by macrophages which promoted cancer progression [37]. Massaro et al. demonstrated that CD47 expression was downregulated in HUVECs by DHA treatment [38]. CD47 causes immune evasion by interaction through expressed SIRPα on myeloid cells which leads to phosphorylation cytoplasmic immuno-receptor tyrosine-based inhibition motifs on SIRPα beside recruitment of Src homology 2 domain-containing tyrosine phosphatases to provoke anti-phagocytic signal [39]. Reduction expression of CD47 has additional support for reinforcement immune responses.

Expression of CD80 and PD-L1 were down-regulated in all combination treatments excluding in Exo-BT under normoxic condition. Talamonti et al. demonstrated that bone marrow-derived M2 macrophages obtained from mice lacking DHA synthesis enzyme Elovl2−/− displayed a notable increase in CD80 and CD86 expression [40]. Based on a meta-analysis study, overexpression of CD80 is associated with growth and metastasis of BC [41]. According to researches, a negative correlation has been discovered between the survival rate of sufferers from BC and the expression of PD-L1 [42]. Fadaee et al. showed that DHA caused down-regulating the expression of PD-L1 in protein level in human colorectal cancer cell lines [43]. Also, Zhang et al. reported that DHA decreased the expression of PD-L1 in lung cancer by rising degradation of ubiquitin-proteasome in vitro and in vivo [44]. CD80 and PD-L1 have essential roles in suppression of immune system so down-regulation of these molecules improve efficacy of therapy.

In this study, decreased expression of co-inhibitory molecules B7-H3 and B7-H4 and increased expression of their regulating miRNA in all groups was experimentally significant. Recent researches have indicated that B7-H3 and B7-H4 are highly expressed in BC tissues compared to normal tissues [18]. These results offer crucial evidence for importance of down-regulation expression of immunosuppressive molecules to conquer malignancy.

About the effect of DHA on miRs, Komi et al. reported that DHA can modulate miRs associated BC. Also, Ghaffari et al. proved that DHA caused increase expression of anti-angiogenic and decrease levels of pro-angiogenic miRs in BC [45]. We evaluated tumor suppressor miRs such as miR-142-3p, miR-30a-5p, miR-133a, miR-194, miR-424, miR-155-5p, miR-143. DHA increased the expression of miR-142-3p and miR-30a-5p, but miR-142-3p did not increased under both conditions in Exo-BT. Expression of miR-30a-5p in DHA-treated cells under hypoxic condition did not increase in MDA-MB-231 and Exo-BT. Compelling evidence indicates that DHA increased miR-133a. Decreased expression of miR-424 was detectable just in Exo-BT. Also, miR-194 was increased except in MDA-MB-231 and Exo-BT. In contrast, Shekari et al. proved that DHA led to a significant under-expression of miR-194 in MKN-45 cell line [46]. Similar to previous studies, we found that miR-155 expression was increased in 3 groups except for the Exo-BT. Also, increased expression of miR-143 in all groups was experimentally significant.

As mentioned in the literature review, both colorectal cancer tissues and cells showed low miR-142-3p expression [47]. MiR-142-3p overexpression inhibits the invasion and migration of colorectal cancer cells [47]. The knockdown or overexpression of miR-30a-5p in the PC-3 cell line impacts cell proliferation, indicating a tumor suppressor function [48]. Compared with control groups, miR-133a expression was significantly decreased in non-small cell lung carcinoma (NSCLC) tissues and cells. MiR-133a overexpression inhibits the viability of A549 cells while miR-133a knockdown enhances their viability [49]. In colon cancer cells, miR-424 was poorly expressed, resulting in malignant progression [50]. There are some studies that show miR-155-5p acts as a tumor suppressor by inhibiting the proliferation and metastasis of melanoma [51] and colorectal cancer cells [52]. A study by Soheilyfar et al. showed that miR-143 reduced BC invasion and metastasis [53].

It has been shown that downregulation of miR-142-3p resulted in upregulation of CD39 in regulatory T (Treg) cells [54], and miR-30a-5p can bind directly to the 3′-untranslated regions (UTR) of CD73 mRNA, subsequently reducing its expression in colorectal, NSCLC and pancreatic cancers [55–57]. Upregulated miR-133a could inhibit proliferation, invasion, and migration and promote cell apoptosis in laryngeal carcinoma by targeting CD47 [58]. MiR-424 has been reported to be inversely correlated with PD-L1 and CD80 expressions in ovarian cancer [59]. Bioinformatics software predicted that miR-155-5p and miR-194-5p can target PD-L1 [60]. Direct target genes of miR-143 are B7-H3 and B7-H4 in colorectal cancer [61].

Contrary to expectations, treatment of BT-474 cell line with DHA showed some conflicting expression especially under normoxic condition (Fig. 5; Table 8). We observed DHA decreased CD47 expression in both cell lines whereas in exosomes alteration of CD47 expression between normoxic and hypoxic conditions didn’t show strike differences. Furthermore, DHA increased miR-133a in Exo-BT. In contrast, exosomal expression of CD47 was down-regulated in two cell lines (Fig. 4; Tables 6 and 7). Further data collection is required to determine why DHA can affect mostly on some factors.

Also, hypoxia induced altered expression level of studied miRs, in some cases relevant to the expression level of target genes. DHA led to the up-regulated cellular expression level of all tumor-suppressor miRs in cells under both normoxic and hypoxic conditions (Fig. 3; Tables 4 and 5). This altered expression pattern was agreement with the expression level of target genes. The exception was the expression of miR-194 in MDA-MB-231 cell line under normoxic condition that was down-regulated after treatment with DHA (Fig. 3; Table 5). Exosomal expression of tumor-suppressor miRs was also up-regulated in MDA-MB-231 cell line after treatment with DHA under both hypoxic and normoxic conditions (Fig. 5; Table 9).

Recommendation of minimum daily consumption of omega-3 PUFAs are 200–450 mg. Also, up to 3 g/day is confirmed by FDA [62]. In clinical settings, enhancement of the immune responses, life quality, and overall survival in cancer patients have been reported as effects of omega-3 PUFAs [62]. Majority of clinical studies in the curative effect of omega-3 PUFAs have demonstrated reduction of C-reactive protein/albumin ratio, platelet aggregation, and inflammatory responses (by decreasing TNF-α, IL-8, and IL-6), increasing of IL-2, improvement immune response, count of neutrophils, helpful modulation of IL-10, decreasing of ICU postoperative, and hospital stay [62]. In palliative management of oral usage of omega-3 fatty acids, studies done for 12–84 days and noted that enhancement of life quality of cancer patients was seen [62]. In randomized clinical trials, omega-3 PUFAs has been employed during range of 3–17 days in the pre and or post-operative setting in the 876 gastrointestinal cancer patients. The findings of these researches were decreasing apoptosis of lymphocytes, and improvement of immune cell like boosting of CD3 + and CD4 + lymphocyte percentage, and reduction of inflammatory responses by decreasing of TNF-α, IL-6, and increasing of IL-10 production [62]. About other application of omega-3 fatty acids in clinical setting, many studies showed that omega-3 fatty acids could increase tolerability and efficacy of chemotherapy. clinical trials that employed DHA alone or combinations of other omega-3 PUFAs in cancer avoidance, and treatment proved DHA could enhance cytotoxic effects of chemotherapeutic drugs [63]. Moreover, DHA supplementation reduced adverse side effects of radio- and chemo-therapy. Combination DHA with cyclophosphamide, epirubicine, and 5-fluorouracil in BC patients caused decreasing of tumor cell proliferation [63]. Also, DHA led to induce apoptosis in the primary cells of CLL patients [64]. About BC, daily consumption of DHA during the chemotherapy in base of DHA levels in RBCs and plasma of BC patients, patients were separated two groups which consist of low and high incorporating. In group of high incorporating, time of tumor development delayed and overall survival longed comparison with the group of low incorporating. In additional, DHA incorporating associates with enzymatic activity, rates of metabolism, background diet, gender, and age of individuals [63].

The achievements of this research have been obtained in ideal laboratory conditions that have a two-dimensional growth environment. While in the clinical situation, cells grow and multiply in a three-dimensional environment, which can be completely different from two-dimensional laboratory conditions. On the other hand, ideal laboratory conditions will not exist in the clinical environment, so clinical results may differ from laboratory results.

**Fig. 6:**
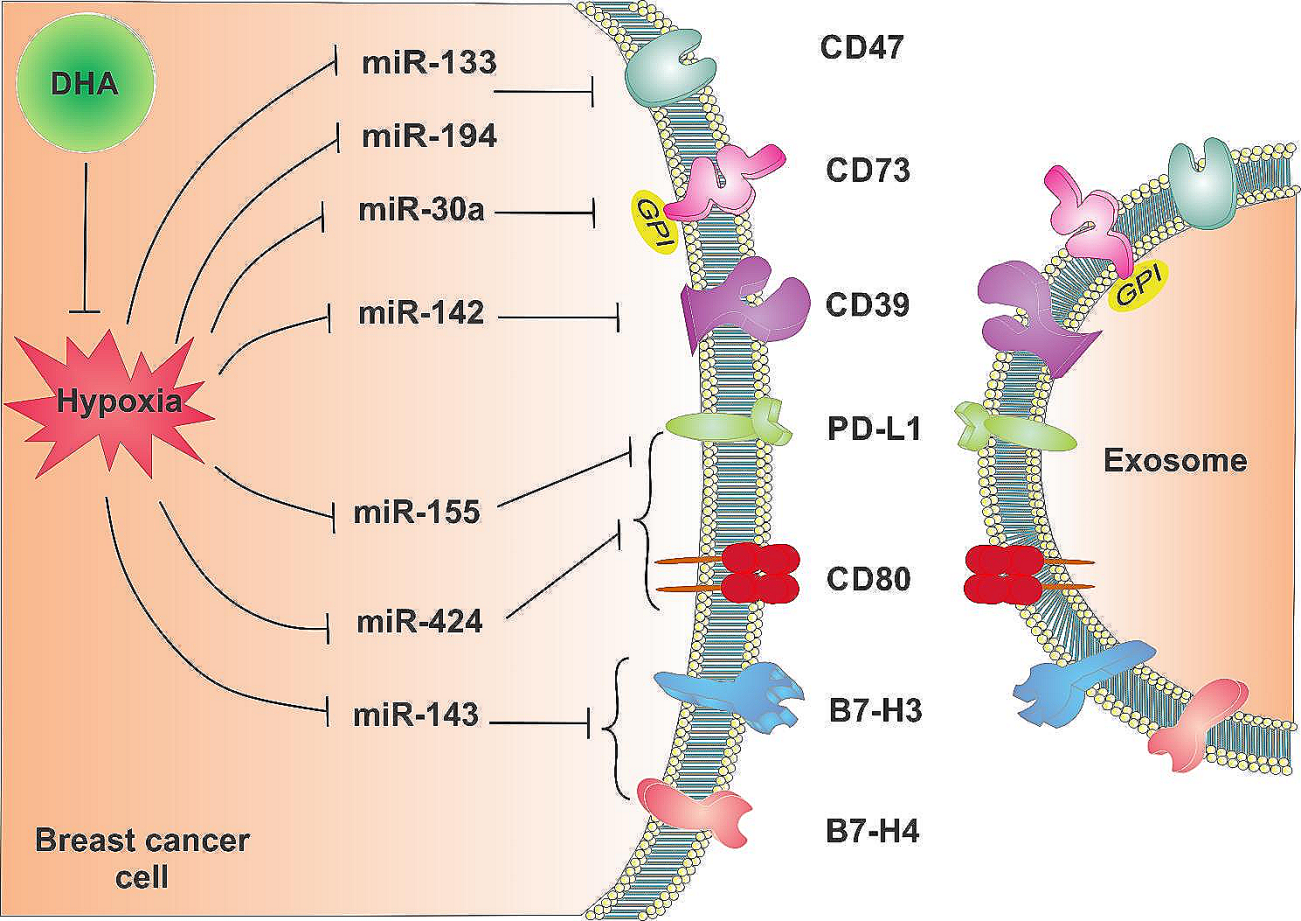
A comprehensive summary of the results

Figure 6 shows a comprehensive summary of all results of this research.

## Conclusions

The up-regulation of immune checkpoint and immunomodulatory molecules, as well as the down-regulation of tumor suppressor miRs under hypoxic conditions, revealed BC progression under these conditions. DHA also reversed hypoxia-induced deregulation of immune-checkpoint and immunomodulatory molecules and their regulatory miRs in BC cells and their derived exosomes. Results of this study provide new insight into DHA’s anti-cancer properties and provide further evidence for the use of DHA as a supplement for BC therapies. Additionally, there is potential for developing new cancer therapeutic strategies through manipulating exosomes and their contents, including miRNAs such as miR-143, miR-155, miR-194, and miR-424, which target multiple immune checkpoints and immunomodulators.

### Electronic supplementary material

Below is the link to the electronic supplementary material.

Supplementary Material 1

## Data Availability

All data generated or analyzed during this study are included in this published article, and its supplementary information files.
