# Supplementary material for: Docosahexaenoic acid (DHA) impairs hypoxia-induced cellular and exosomal overexpression of immune-checkpoints and immunomodulatory molecules in different subtypes of breast cancer cells
Source: BMC Nutr. 2024 Mar 4;10:41. doi: 10.1186/s40795-024-00844-y (PMC10910708; doi:10.1186/s40795-024-00844-y)
Supplement: Supplementary file 1 — Supplementary Material 1 [file 40795_2024_844_MOESM1_ESM.doc]

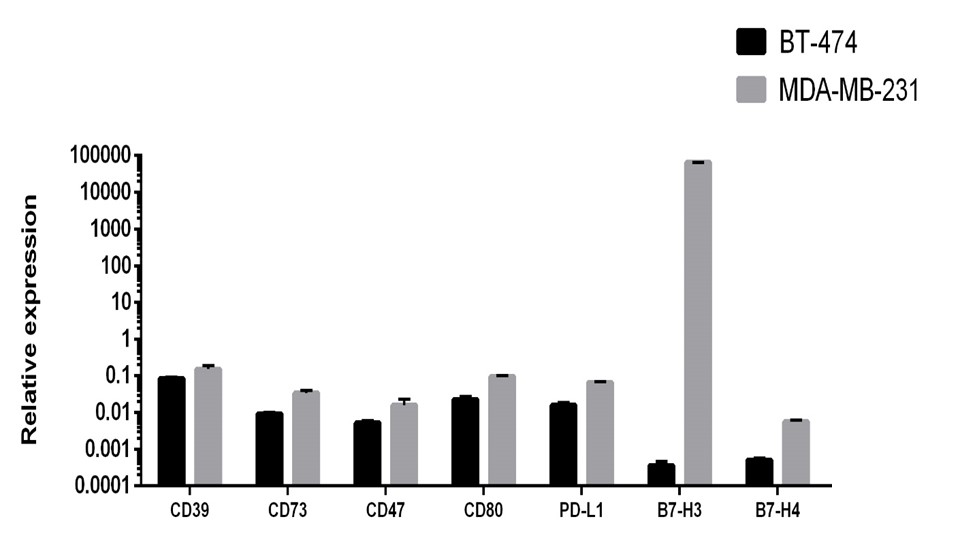


**Supplementary figure 1- Expression levels of target genes in untreated BC cell lines**


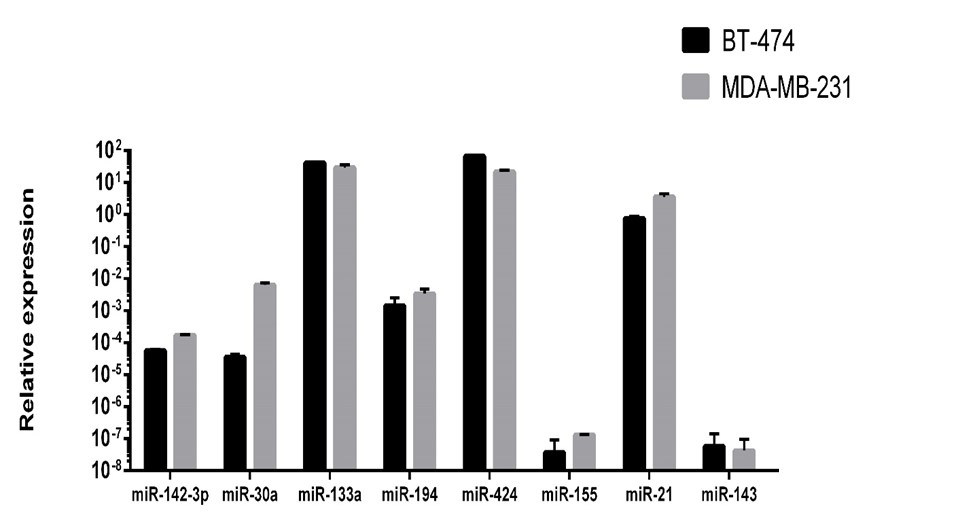


**Supplementary figure 2- Expression levels of studied miRs in untreated BC cell lines**


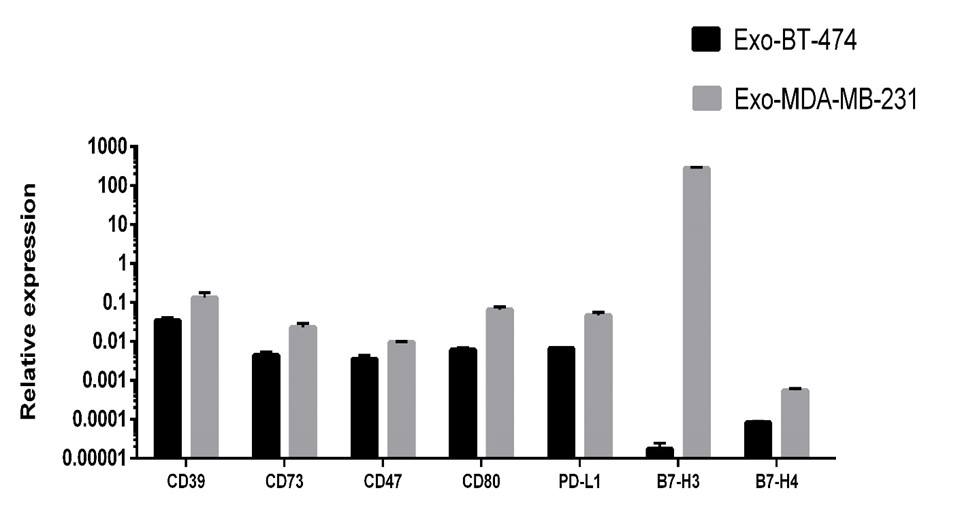


**Supplementary figure 3- Expression levels of target genes in untreated exosomes-derived from BC cell lines**


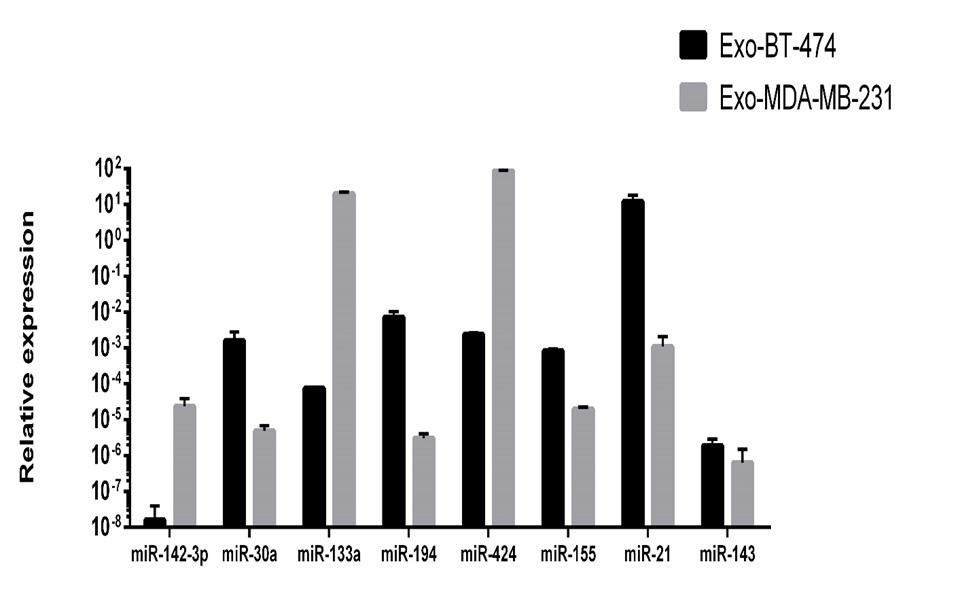


**Supplementary figure 4- Expression levels of studied miRs in untreated exosomes-derived from BC cell lines**

**
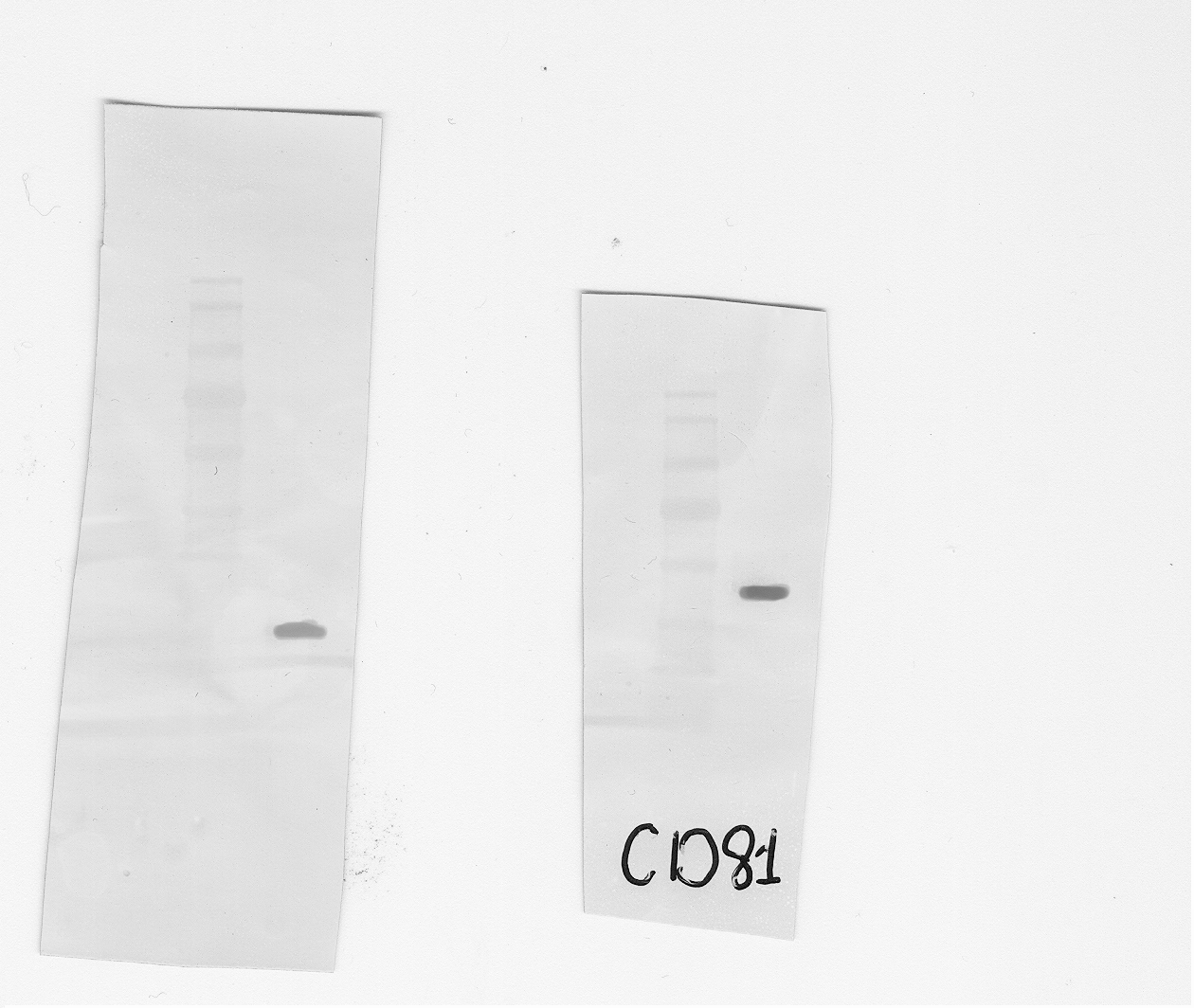
**


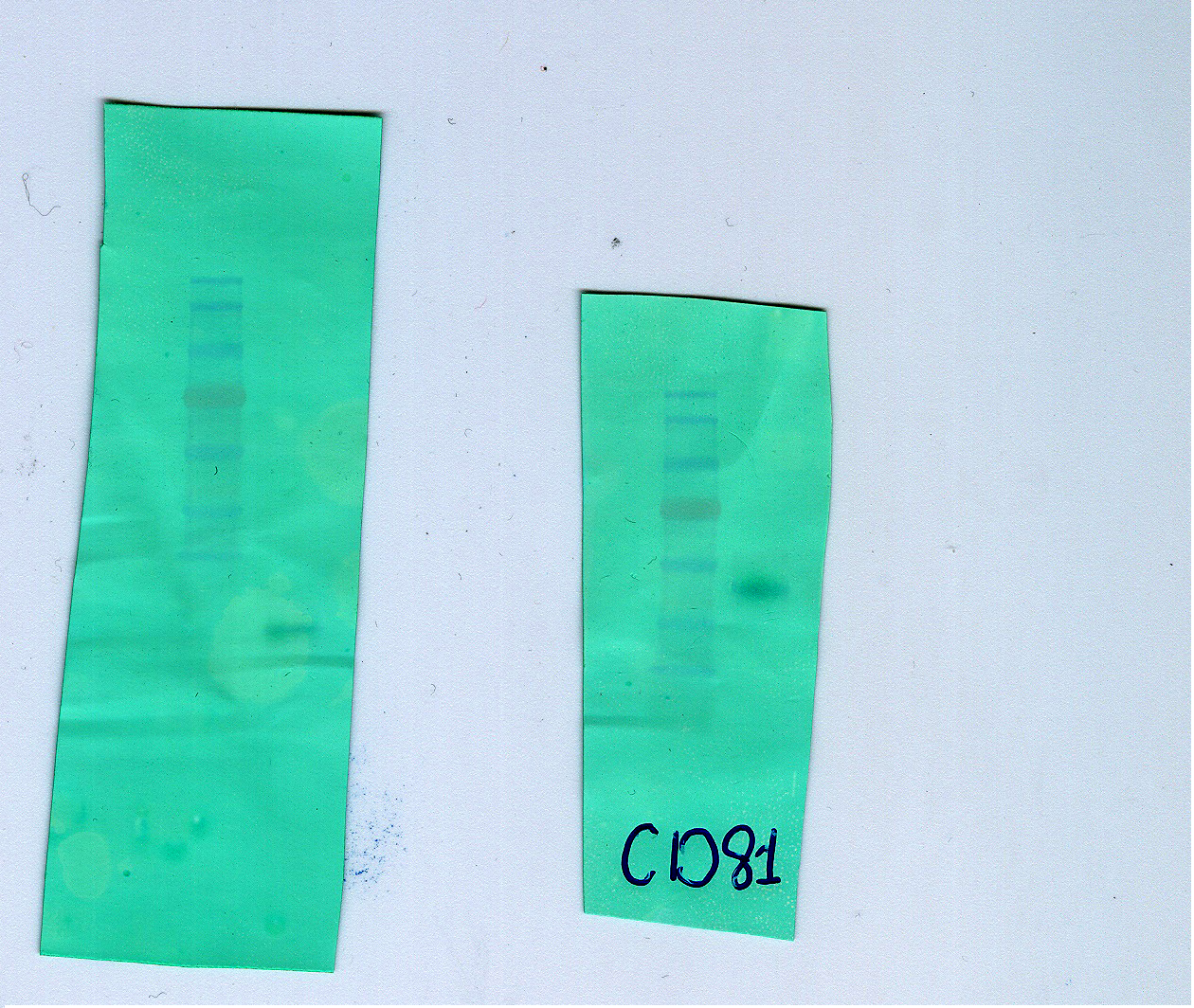


**Supplementary figure 5-** Western blotting result of expression levels of CD9, and CD81.

Because the sample size (number of samples) in this research is small, we used a small gel to obtain best results from western blot, so this image is the whole gel. In other words, the gel used in this research is a mini size gel.


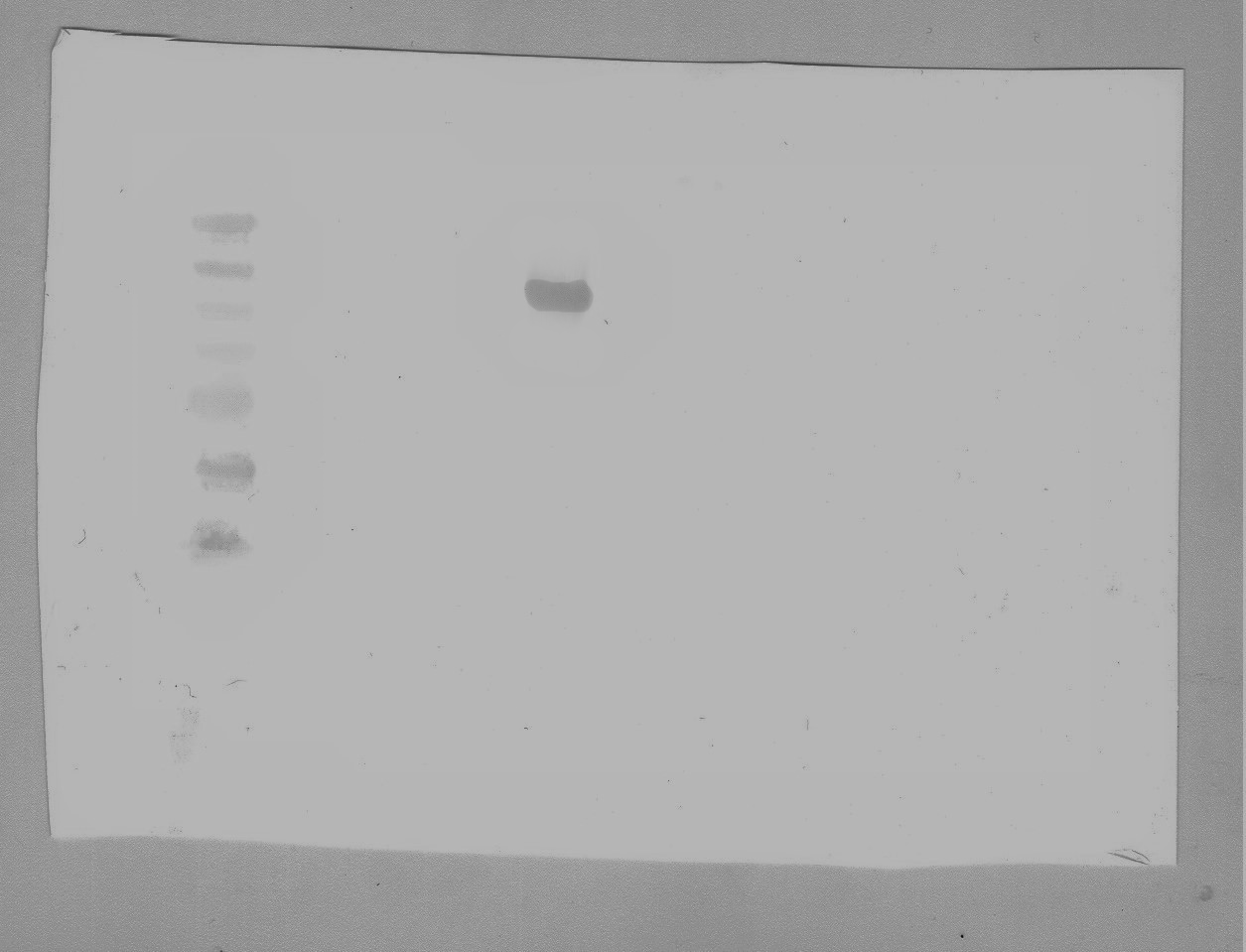


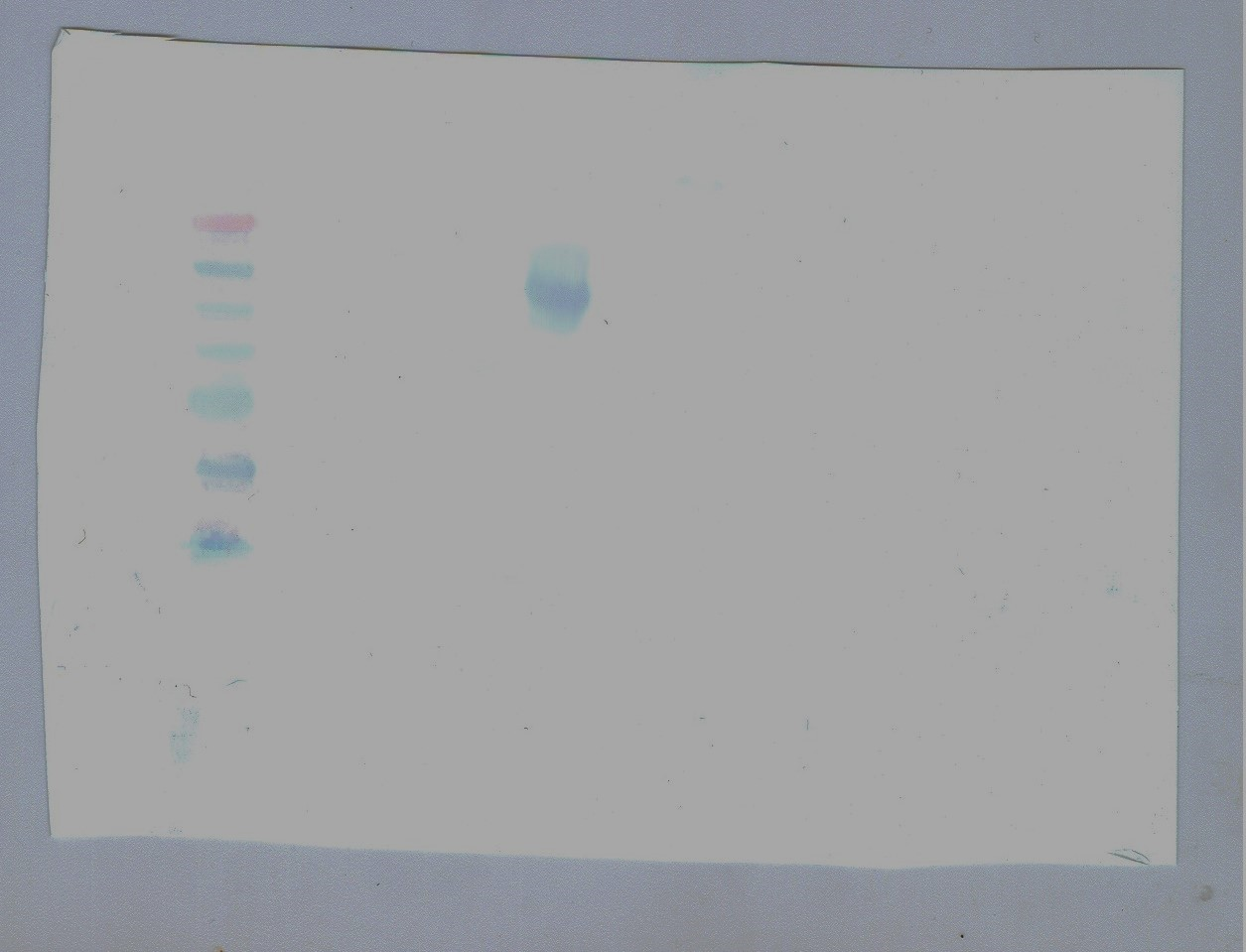


**Supplementary figure 6-** Western blotting result of expression level of CD63.

A mini size gel was used in this research to obtain best results from western blot. This image is the whole gel.


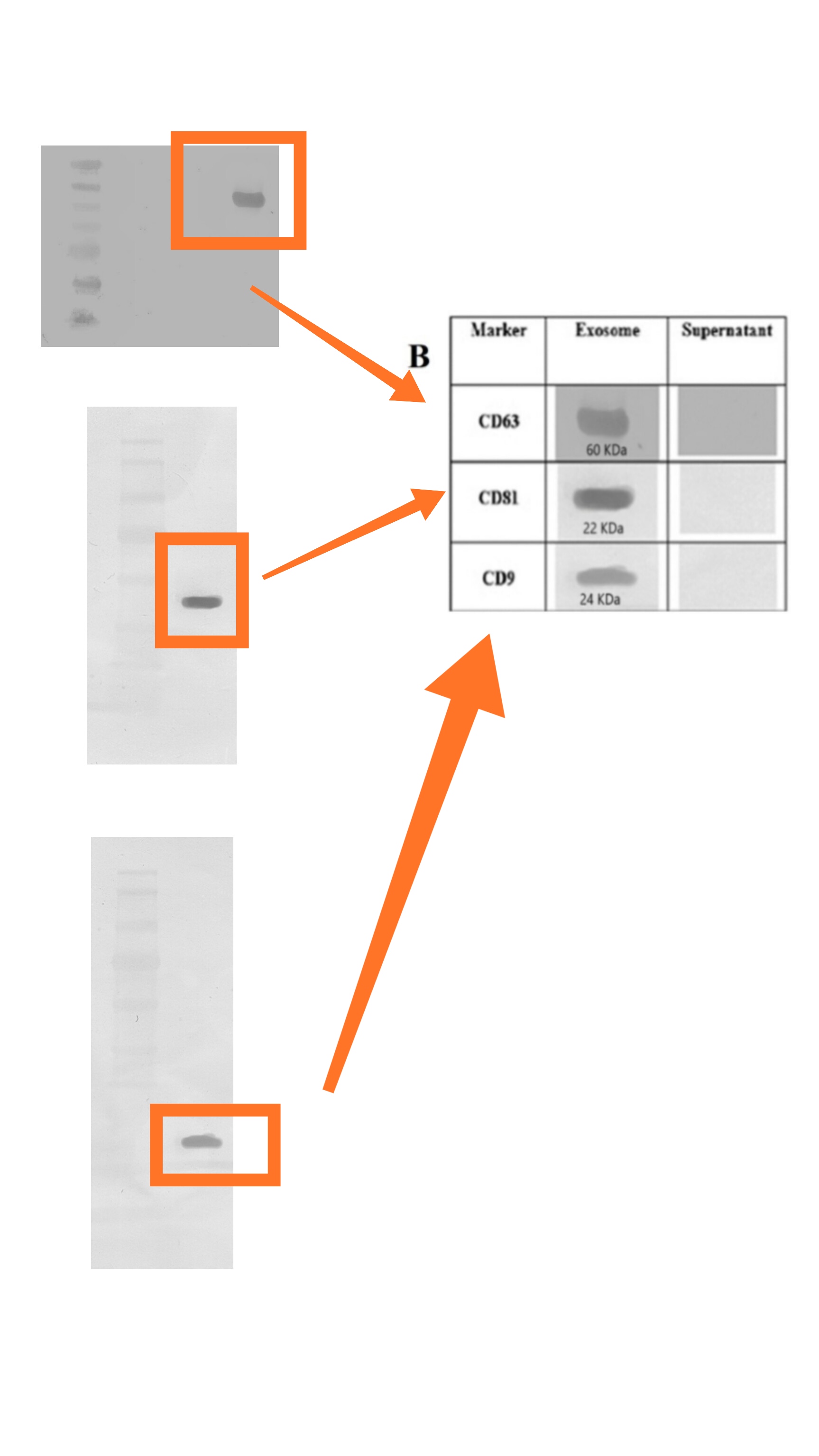


**Supplementary figure 7-** grouping sources of Figure 1.
